# Insights into the Evolutionary Origin of Mediterranean Sandfly Fever Viruses

**DOI:** 10.1128/mSphere.00598-20

**Published:** 2020-09-02

**Authors:** Marco Marklewitz, David P. Tchouassi, Christian Hieke, Verena Heyde, Baldwyn Torto, Rosemary Sang, Sandra Junglen

**Affiliations:** a Institute of Virology, Charité—Universitätsmedizin Berlin, corporate member of Free University Berlin, Humboldt-University Berlin, and Berlin Institute of Health, Berlin, Germany; b German Center for Infection Research, Berlin, Germany; c International Centre of Insect Physiology and Ecology, Nairobi, Kenya; d Center for Virus Research, Kenya Medical Research Institute, Nairobi, Kenya; U.S. Centers for Disease Control and Prevention

**Keywords:** *Phenuiviridae*, phlebovirus, sandfly, arbovirus, Kenya

## Abstract

Sandfly-transmitted phleboviruses (family *Phenuiviridae*, order *Bunyavirales*) are associated with febrile illness and infections of the nervous system in humans. These viruses are almost exclusively found in tropical areas of the New World and restricted to semiarid and temperate zones in the Old World. Here, we discovered seven strains of four previously unknown phleboviruses, named Bogoria virus (BOGV), Embossos virus (EMRV), Kiborgoch virus (KBGV), and Perkerra virus (PERV), as well as the recently discovered Ntepes virus, in sandflies collected in the Kenyan Rift Valley. The genomes have a tripartite organization with conserved termini typical of phleboviruses. LOBV, PERV, and EMBV showed low similarity to known phleboviruses, with less than 55% pairwise amino acid identities in the RNA-directed RNA polymerase (RdRp) proteins, and defined a highly diversified monophyletic clade in sister relationship to the sandfly fever Sicilian serocomplex. All three viruses failed to react with sandfly fever Sicilian virus antisera in recombinant immunofluorescence assays (rIFA), suggesting that the viruses belong to a yet-unknown serogroup. In contrast, KBGV was closely related to Toscana virus (84% identity of RdRp proteins) and shared a most recent common ancestor with the clade comprising sandfly fever Naples and Toscana viruses. KBGV reacted with sandfly fever Naples and Toscana virus antisera in rIFA. The genetic diversity of the detected viruses and their phylogenetic positions implies that the Old World sandfly-borne phleboviruses originated from sub-Saharan Africa. Importantly, our findings suggest that diseases associated with sandfly-borne phlebovirus infections may also affect the Kenyan population.

**IMPORTANCE** Studies on the genetic diversity of arthropod-borne viruses circulating in rural regions can provide critical early indications on new emerging viruses essential for global epidemic preparedness. In this study, we describe the discovery of four phleboviruses in sandflies from the Kenyan Rift Valley. The novel viruses are related to the two medically important serocomplexes, sandfly fever Naples and sandfly fever Sicilian, that are associated with febrile illness and neuroinvasive infections and which were previously not known to occur in sub-Saharan Africa. Knowledge on the occurrence of sandfly-borne phleboviruses in Kenya and elsewhere in Africa can help to decipher their contributions in the etiologies of fevers of unknown origin in patients. Our findings on five genetically diverse phleboviruses detected in Kenya suggest that the common ancestor of Old World phleboviruses existed in sub-Saharan Africa, a hot spot for emerging arboviruses.

## Abstract (precis)

Studies on the genetic diversity of arthropod-borne viruses circulating in rural regions can provide critical early indications on new emerging viruses essential for global epidemic preparedness. In this study, we describe the discovery of four phleboviruses in sandflies from the Kenyan Rift Valley. The novel viruses are related to the two medically important serocomplexes, sandfly fever Naples and sandfly fever Sicilian, that are associated with febrile illness and neuroinvasive infections and which were previously not known to occur in sub-Saharan Africa. Knowledge on the occurrence of sandfly-borne phleboviruses in Kenya and elsewhere in Africa can help to decipher their contributions in the etiologies of fevers of unknown origin in patients. Our findings on five genetically diverse phleboviruses detected in Kenya suggest that the common ancestor of Old World phleboviruses existed in sub-Saharan Africa, a hot spot for emerging arboviruses.

## INTRODUCTION

Sandfly-borne phleboviruses (family *Phenuiviridae*, order *Bunyavirales*) of the Old World have mainly been reported in the Mediterranean area, in North Africa, and in Western and Central Asia (reviewed in reference 1). These viruses are exclusively transmitted by sandflies of the genera *Phlebotomus* and *Sergentomyia* in the Old World and by species of the genus *Lutzomyia* in the New World. Infections with sandfly-borne phleboviruses can lead to fever, nausea, vomiting, febrile illness, and central nervous system manifestations. Antibodies against sandfly-borne phleboviruses are widely distributed in humans and animals in the above-mentioned geographic regions, underlining the public health importance of these infections (2, 3).

The most important medically relevant sandfly-borne phleboviruses in the Old World are Toscana virus, sandfly fever Naples virus, and sandfly fever Sicilian virus, which belong to the serological complexes sandfly fever Naples serocomplex (comprising Toscana, sandfly fever Naples, and related viruses) and sandfly fever Sicilian serocomplex (comprising sandfly fever Sicilian virus and related viruses). Toscana virus is the only sandfly-borne phlebovirus which has a neurotropism; it typically causes meningitis and encephalitis, but other neuroinvasive manifestations, such as Guillain-Barré syndrome, hydrocephalus, myositis, fasciitis, polymyeloradiculopathy, deafness, and facial paralysis, have also been reported (4). Toscana virus circulates widely in European countries around the Mediterranean basin (5), as well as in Algeria, Djibouti, Morocco, and Tunisia in Northern Africa (6, 7). Symptoms of sandfly fever Naples virus and sandfly fever Sicilian virus infections are clinically similar and often described as 3-day-fever characterized by abrupt onset of fever, headache, muscular pain, photophobia, and nausea (1). Both viruses are endemic in the Mediterranean region and western and central Asia (2). In Africa, seroprevalence of sandfly fever Naples virus has been described in Morocco, Algeria, Egypt, Sudan, Djibouti, and Ethiopia (2, 7). Sandfly fever Sicilian virus is endemic to the same countries in Africa as sandfly fever Naples virus except Djibouti and Ethiopia but has been detected additionally in Uganda and Somalia (2, 8). In 2011, sandfly fever Sicilian virus was identified as responsible for an outbreak of febrile illness in Ethiopia (9). However, most data on virus distribution in these African countries are based on serosurveillance studies conducted during the 1980s without genetic characterization of the causative viruses. Historically, sandfly fever Naples and Sicilian viruses have been of great importance for military conquests starting from the beginning of the 18th century up to the Second World War, where huge outbreaks occurred in nonimmune persons (10–12).

Members of the sandfly fever Naples serocomplex are classified into the species *Gordil phlebovirus*, *Massilia phlebovirus*, *Naples phlebovirus*, *Punique phlebovirus*, *Saint Floris phlebovirus*, *Tehran phlebovirus*, *Toscana phlebovirus*, and *Zerdali phlebovirus* by the International Committee on Taxonomy of Viruses (ICTV). The sandfly fever Sicilian serocomplex comprises the ICTV-classified species *Dashli phlebovirus*, *Toros phlebovirus*, and *Sicilian phlebovirus*. Several additional phleboviruses that group in phylogenetic analyses together with Toscana virus and sandfly fever Naples virus have been described. Whereas the majority of these viruses were detected in sandflies from the Mediterranean, e.g., Massilia virus in Portugal and France or Punique virus in Tunisia, two distantly related viruses, namely, Gordil virus and Saint Floris virus, were isolated from rodents in the Central African Republic in 1971 (13). Antibodies against the two latter viruses were detected in human sera from Somalia and Sudan (2). Antibodies against Saint Floris virus were also found in Egypt. Serum samples collected from sheep in Burkina Faso showed reactivity against Gordil virus and Saint Floris virus, suggesting a widespread circulation (14). To date, both viruses have not been associated with human disease. Two additional viruses, Dashli virus and Toros virus, form a monophyletic clade together with sandfly fever Sicilian virus. Dashli virus has been isolated from sandflies in the genus *Sergentomyia* in Iran (15) whereas Toros virus was initially isolated from sandflies collected in Turkey (16). Similarly, both viruses have not been associated with disease in humans, thus far. Viruses of the Salehabad serocomplex were considered not to infect humans or domestic animals for a long time. However, recent studies have found antibodies against Adana virus belonging to this complex in humans, goats, sheep, and dogs in Turkey, and Adria virus was detected in a febrile child from Greece (17, 18).

We recently detected a previously unknown phlebovirus termed Ntepes virus (NPV) in sandflies from Kenya (19). Neutralizing antibodies against NPV were found in humans in two distant geographic areas (>600 km apart) in Kenya, suggesting a wider distribution of the virus. Despite the clinical relevance of sandfly-borne infections and the high abundance of sandflies in sub-Saharan African countries, NPV is so far the southernmost described sandfly-borne phlebovirus from a tropical savanna climate. In this study, we sought to determine whether further phleboviruses circulate in sandflies and humans in Kenya.

## RESULTS

### Detection of phleboviruses in sandflies.

In 2015 and 2016, 3,958 phlebotomine sandflies were collected in and around households in the villages of Ntepes and Kapkuikui from dusk until dawn (Table 1). Specimens were combined into 400 pools and subsequently into 40 superpools. From four superpools, sequence fragments were obtained which showed ca. 47 to 55% pairwise nucleotide identities to the RNA-directed RNA polymerase (RdRp) gene of phleboviruses and 53 to 78% among themselves. In addition, NPV was detected in one superpool. Individual pools of the six positive superpools were screened by virus-specific real-time reverse transcription-PCRs (RT-PCRs), revealing seven strains of five distinct viruses (Table 2). The newly detected viruses were named after geographic references in the area of Baringo County where the phlebovirus-positive sandfly specimens were collected: Bogoria virus (BGRV), named after Lake Bogoria; Embossos virus (EMBV), named after the Embossos River; Perkerra virus (PERV), named after the Perkerra River; and Kiborgoch virus (KBGV), named after the Kiborgoch Community Wildlife and Wetlands Conservancy south of Marigat subcounty.

**TABLE 1:** Sandflies collected in Marigat district using CDC miniature light traps baited with different light sources inside homes (indoors) and outside (outdoors)

Light type	Locality	Situation	No. of traps/12 h	No. of sandflies
Incandescent	Kapkuikui	Indoors	2	827
		Outdoors	1	1,430
	Ntepes	Indoors	1	93
		Outdoors	2	18
Ultraviolet	Kapkuikui	Outdoors	1	336
	Ntepes	Outdoors	2	198
Ultraviolet + CO_2_	Ntepes	Outdoors	1	99
Red	Kapkuikui	Outdoors	1	25
	Ntepes	Outdoors	1	24
Green	Kapkuikui	Outdoors	2	333
Blue	Ntepes	Outdoors	1	113
Red/green/blue (RGB)	Kapkuikui	Outdoors	1	414
	Ntepes	Outdoors	1	48
Total			17	3,958

**TABLE 2:** Novel phleboviruses detected in this study

Virus	Strain	Locality	No. of RNA copies/ml	Segment and accession no.
Embossos virus	SP288/KE/2016	Kapkuikui	9.12 × 10^7^	L: MT270825
				M: MT270826
				S: MT270827
	SP394/KE/2016	Kapkuikui	1.77 × 10^6^	S: MT625967^*a*^
				
Bogoria virus	SP105/KE/2016	Kapkuikui	7.79 × 10^5^	L: MT270828
				M: MT270829
				S: MT270830
				
Kiborgoch virus	SP381/KE/2016	Kapkuikui	8.77 × 10^8^	L: MT270831
				M: MT270832
				S: MT270833
				
Perkerra virus	SP166/KE/2016	Kapkuikui	1.61 × 10^8^	L: MT270834
				M: MT270835
				S: MT270836
	SP162/KE/2016	Kapkuikui	9.91 × 10^8^	S: MT625968^*a*^
				
Ntepes virus	SP375/KE/2016	Kapkuikui	2.96 × 10^8^	L: MT625964
				M: MT625965
				S: MT625966

a Partial.

To estimate which type of sandflies were present in the virus-positive pools, a fragment of the invertebrate *COI* gene was amplified, and 10 clones were sequenced from each virus-positive sandfly pool (Table 2). Sergentomyia schwetzi sandflies were found in every sandfly pool with the exception of the EMBV-positive sample SP394, for which no species association was possible due to low sequence identity (maximum identity of 97.1% to Sergentomyia bedfordi). Of note, sequences for which no clear species association was possible were also detected in the PERV-positive pools SP162 and SP166, the EMBV-positive pool SP288, the NPV-positive pool SP375 and the KBGV-positive pool SP381. In addition to *S. schwetzi*, Sergentomyia inermis sandflies were identified in the EMBV-positive pool SP288 and Sergentomyia dreyfussi in the NPV-positive pool SP375.

We further sought to identify the vertebrate sources on which the virus-positive sandflies had fed using a PCR targeting the vertebrate *COI* gene. Sequencing of the amplicons of sandfly pools SP105 (positive for BGRV) and SP166 (positive for PERV) targeting the *COI* gene resulted in high similarities to the mitochondrial genome of humans (SP105, 98.6% Homo sapiens; SP166, 98.9% Homo sapiens) and sequencing of SP381 (positive for KBGV) showed high similarity to cattle (98.7% Bos taurus) in GenBank database searches. No amplicon was obtained from the remaining virus-positive samples (SP162, SP288, SP375, and SP394) (Table 2).

### Genome sequencing and analyses.

**FIG 1:**
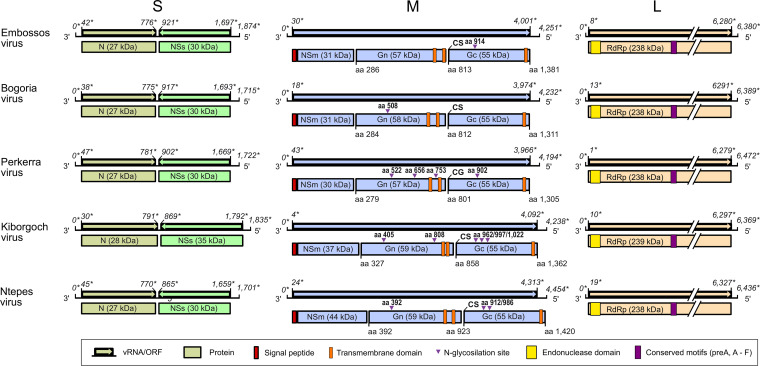
Genome organization of Embossos virus, Bogoria virus, Perkerra virus, Kiborgoch virus, and Ntepes virus. Black lines represent genome segments, arrows represent open reading frames, and boxes represent translated proteins. Asterisks next to genome positions indicate relative genome positions.

**FIG 2:**
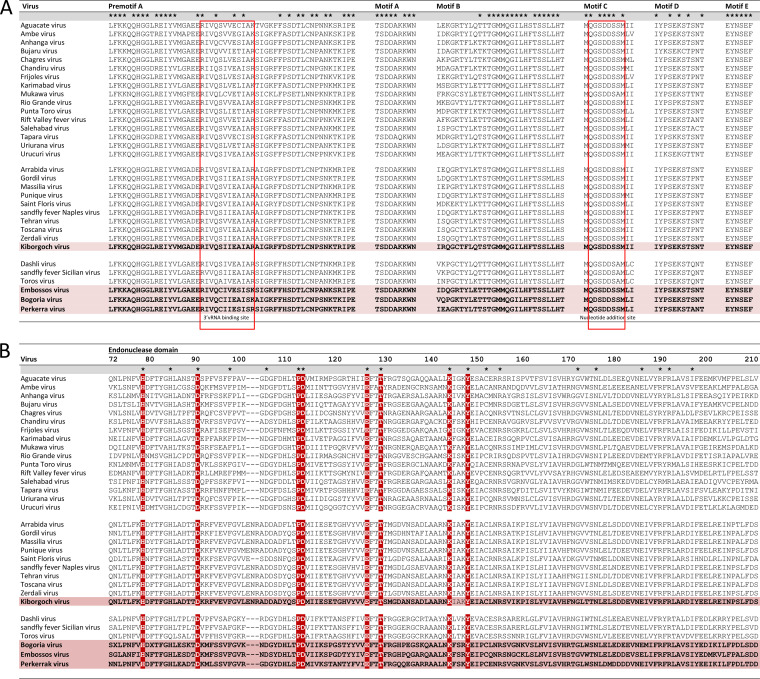
Conserved RdRp palm motifs and endonuclease domains of phleboviruses and viruses sequenced in this study. Sequence alignments of the highly conserved motifs within the phleboviral RdRp protein (A) and of the endonuclease domain (B) of phleboviruses and EMBV, BGRV, PERV, and KBGV. Residues conserved throughout all taxa are marked with an asterisk. Highly conserved endonuclease residues are highlighted in red.

The genomes of BGRV, EMBV, KBGV, and PERV were sequenced directly from the phlebovirus positive sandfly homogenates by high-throughput sequencing (HTS). Sequence gaps were closed using seminested RT-PCR, resulting in complete coding sequences (CDS) and almost complete noncoding regions. All viruses showed a tripartite genome organization comprising a large (L), a medium (M), and a small (S) segment (Fig. 1). The L segments of BGRV, EMBV, KBGV, and PERV each have a single open reading frame (ORF) 6,273, 6,279, 6,273, and 6,288 nucleotides (nt) in length, respectively. The transduced amino acid sequence of the KBGV L ORF showed maximum pairwise identity to the RdRp protein of Toscana virus (85%), whereas the ORFs of BGRV, EMBV, and PERV revealed maximum pairwise identities of 59% to the RdRp protein of sandfly fever Sicilian virus. The RdRp protein palm motifs, namely, pre-A motif and motifs A to E, which are highly conserved among phleboviruses, were identified in all four viruses (Fig. 2). The N-terminal region of the bunyavirus RdRp contains an endonuclease domain that facilitates a cap-snatching mechanism typical for negative-sense RNA viruses (20). The characteristic conserved residues (H…D…PD…ExT…K) that are responsible for the cation binding and the catalytic activity of the endonuclease were conserved in the RdRp proteins of BGRV, EMBV, KBGV, and PERV (Fig. 2). The M segments of BGRV, EMBV, PERV, and KBGV contained single ORFs of 3,972, 3,957, 3,924, and 4,092 nt, respectively. The transduced amino acid sequences showed similarities of 43 to 59% to the glycoprotein precursor protein (GPC) of phleboviruses. The phlebovirus GPC is posttranslationally cleaved into the glycoproteins Gn and Gc and the nonstructural protein NSm. These cleavage products were identified in BGRV (58, 55, and 31 kDa), EMBV (57, 55, and 31 kDa), PERV (57, 55, and 30 kDa), and KBGV (59, 55, and 37 kDa) using the pfam database (https://pfam.xfam.org) (Fig. 1). The S segments of BGRV, EMBV, PERV, and KBGV contained two ORFs in an ambisense orientation. The transduced 3′-terminal ORF showed similarities of 53 to 88% to the nucleocapsid protein (N) of phleboviruses, and the transduced 5′ ORF showed highest similarities to the nonstructural (NSs) protein of phleboviruses of 26 to 52%.

According to the phlebovirus species demarcation criteria of the ICTV, unique species show more than 5% distance in their RdRp protein sequences (21). The detected viruses showed at least 15% genetic distance in their RdRp proteins to other phleboviruses (KBGV, <15%; BGRV, EMBV, and PERV, <41%), indicating that these viruses represent four novel species in the genus *Phlebovirus*. A related taxonomic proposal has been submitted to the ICTV.

To test for intraspecies diversity, we sequenced the N genes of EMBV and PERV based on virus-specific primers flanking the N ORF. We observed a single nonsynonymous substitution for EMBV (_233_Glu to _233_Leu) and various synonymous nucleotide substitutions randomly distributed across the N genes: four for EMBV and five for PERV.

In addition, the entire CDS of NPV was directly sequenced from the sandfly homogenate and compared to the strain initially sequenced from infectious cell culture supernatant originating from sandflies collected in the same geographic region in 2014 (19). For the RdRp, GPC, N, and NSs genes, 15, 13, 2, and 4 synonymous substitutions, respectively, were detected between the two strains. Corresponding nucleotide identities of the two respective CDS were 99.7% (RdRp), 99.6% (GPC), 99.7% (N), and 99.5% (NSs). One nonsynonymous nucleotide substitution each was detected in the RdRp (_508_Ser to _508_Asp), GPC (_433_Lys to _433_Arg), and NSs (_147_Val to _147_Ala) genes of the newly sequenced NPV strain SP375-KE-2016 derived directly from sandflies. No nonsynonymous nucleotide substitution was detected in the N gene.

### Phylogenetic relationship.

**FIG 3:**
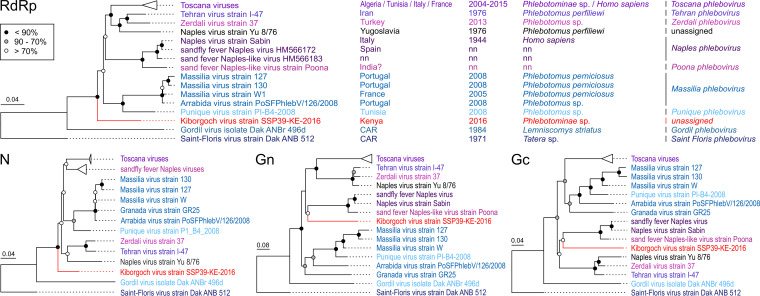
Phylogenetic analyses of Kiborgoch virus and phleboviruses. Maximum-likelihood phylogenetic analyses of the entire RdRp, N, Gn, and Gc protein sequences were inferred in PhyML using a MAFFT alignment, LG substitution model, and 1,000 bootstrap replicates. The virus sequenced in this study is shown in red. Bootstrap support values are represented by circles at the respective nodes, categorized as <95% (black), 90 to 70% (gray), or >70% (white).

**FIG 4:**
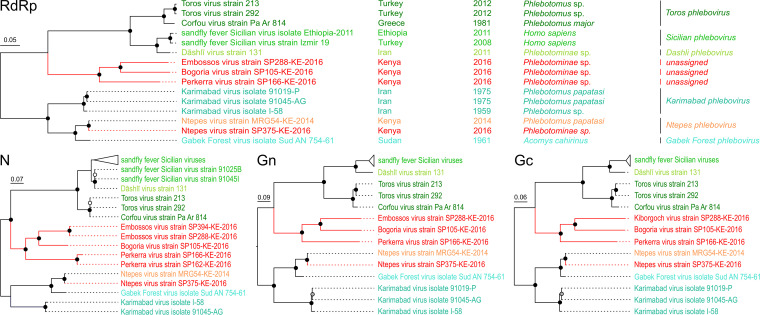
Phylogenetic analyses of Embossos virus, Bogoria virus, Perkerra virus, and Ntepes virus. Maximum-likelihood phylogenetic analyses of the entire RdRp, N, Gn, and Gc protein sequences were inferred in PhyML using a MAFFT alignment, LG substitution model, and 1,000 bootstrap replicates. The viruses sequenced in this study are shown in red. Bootstrap support values are represented by circles at the respective nodes, categorized as <95% (black), 90 to 70% (gray), or >70% (white).

Phylogenetic analyses based on the RdRp proteins consistently showed that BGRV, EMBV, and PERV form a diversified monophyletic clade in sister relationship to the clade comprising the sandfly fever Sicilian viruses, Dashli virus, and Toros virus (Fig. 3), whereas KBGV was placed in a basal position to the clade comprising Toscana virus and sandfly fever Naples virus, among others (Fig. 4). The NPV strain grouped with the previously detected NPV strain (19). Further analyses based on either the Gn, Gc, and N nucleotide or protein sequences of the novel viruses and their closest relatives confirmed the findings obtained for the RdRp-based trees (Fig. 3 and 4). However, KBGV was placed as a sister taxon to sandfly fever Naples virus in phylogenetic analyses based on Gn and Gc protein sequences, albeit with low support values.

### Antigenic relationship.

**FIG 5:**
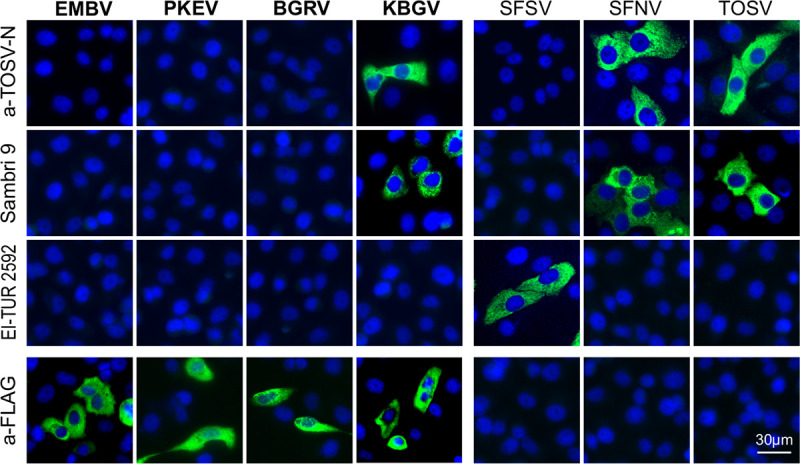
Recombinant immunofluorescence assay of Embossos virus, Bogoria virus, Perkerra virus, and Kiborgoch virus and phlebovirus serocomplexes. Cells either overexpressed FLAG-tagged full-length N genes of EMBV, BOGV, PERV, and KBGV or were infected with sandfly fever Sicilian virus (SFSV), sandfly fever Naples virus (SFNV), and Toscana virus (TOSV) and were subsequently incubated with human serum samples reactive against SFSV, SFNV, and TOSV to investigate serological cross-reactivity.

Attempts to isolate the viruses in cell culture using cell lines derived from sandflies, mosquitoes, and nonhuman primates failed. Thus, we used synthesized N-gene constructs of EMBV, BGRV, KBGV, and PERV to establish recombinant immunofluorescent assays (rIFA) to test for cross-reactivity of the newly detected viruses with related serogroups. Incubation of the Toscana virus N antiserum with overexpressed KBGV N showed prominent reactivity, whereas BGRV N, EMBV N, and PERV N did not react with the antiserum (Fig. 5). These results were confirmed, as a human serum sample (Sambri 1) reactive against sandfly fever Naples virus and Toscana virus also showed reactivity against KBGV N but not against expressed BGRV N, EMBV N, and PERV N proteins (Fig. 5). In addition, a second human serum sample (EI-TUR 2592) containing antibodies against sandfly fever Sicilian virus did not react with any of the new viruses but did react with sandfly fever Sicilian virus (Fig. 5). Taken together, these findings suggest that KBGV may belong to the sandfly fever Naples/Toscana virus serogroup, whereas BGRV, EMBV, and PERV seem to establish a new serogroup tentatively named the Marigat serogroup.

### Screening of human serum samples.

Extracted RNA of individual human serum samples (*n* = 244) collected from patients from Marigat subcounty with fevers of unknown origin were investigated by specific real-time RT-PCR to probe for direct evidence of the virus in human blood. All serum samples tested negative for BGRV, EMBV, PERV, and KBGV. Unfortunately, testing for the presence of antibodies against the four viruses was not carried out due to the paucity of material.

## DISCUSSION

Sandfly-borne phleboviruses of the Old World are so far limited to semiarid and temperate regions, e.g., the Mediterranean, North Africa, India, and western and central Asia. Here, we describe the discovery of four previously unknown phleboviruses (BGRV, EMBV, PERV, and KBGV), as well as the detection of NPV in sandflies from an area with tropical savanna climate in sub-Saharan Africa. The viruses are distantly related to the medically important sandfly fever Sicilian serocomplex (BGRV, EMBV, and PERV), sandfly fever Naples serocomplex (KBGV), and Karimabad serocomplex (NPV), indicating the circulation of taxonomically highly diverse sandfly-borne phleboviruses in Kenya. We recently described the discovery and isolation of NPV from sandflies collected in Ntepes village, Marigat district, in 2014 (19). The repeated detection of NPV in sandflies originating from the same geographic area 2 years after the initial detection, together with our previous findings on the presence of neutralizing antibodies against NPV in humans from different regions in Kenya, provides further evidence that the virus is endemic and widely circulating in the country.

BGRV, EMBV, PERV, and KBGV were exclusively detected in sandflies collected in one of the two sampling locations, in the village Kapkuikui (Table 1). BGRV, EMBV, and KBGV were found in sandflies trapped outside human dwellings, whereas PERV was detected in sandflies collected inside homes. Given the limited flight range of sandflies (22), our findings suggest a direct risk of exposure of humans to the four viruses (22). Further, we showed by blood meal analyses that the sandflies of the BGRV- and PERV-positive pools had fed on humans. The sandflies of the KBGV-positive pool were found to have fed on cattle. About 11 to 17 sandfly species are known to occur in the Marigat region, with *Sergentomyia schwetzi* being the most abundant species reported to feed on humans, cows, goats, and rabbits (23–25). However, since pooled specimens (*n* = 10) were used in these analyses, the blood meal sources may also stem from a sandfly that was not infected with any of the viruses. Further studies aiming at the identification of the vertebrate host and sandfly species involved in maintenance of BGRV, EMBV, PERV, and KBGV will be key to understanding the ecology of these viruses. In addition, studies involving testing of human sera are needed to identify if humans can be infected with the newly detected viruses and whether infections are associated with symptoms of disease.

In the absence of data on virus isolation, preliminary serological investigations were conducted using rIFA based on expressed N proteins. None of the N proteins of BGRV, EMBV, and PERV reacted with antisera against the phylogenetic related group of sandfly fever Sicilian viruses or with antisera against the sandfly fever Naples serocomplex and Toscana virus N protein, suggesting that the three viruses belong to a previously unknown serogroup. The absence of serological cross-reactivity of BGRV, EMBV, and PERV with the known sandfly-borne serogroups may have prevented earlier detection of sandfly-borne phleboviruses in Kenya. Although KBGV showed reactivity with antisera against the sandfly fever Naples serocomplex and Toscana virus N protein, antibodies against the latter two have so far not been identified in Kenya. Antibodies against the sandfly fever Naples serocomplex were found in Ethiopia and Djibouti but not in Somalia, Senegal, Liberia, Kenya, and Sudan in the 1970s (2, 9).

Species of sandflies of the genus *Sergentomyia* have been suggested to be involved in the transmission of NPV (19). Our present study confirms that the *Sergentomyia* sandflies might be associated with NPV. *Sergentomyia schwetzi* and *Sergentomyia dreyfussi* sandflies have been detected in the NPV-positive sandfly pool SP375. In addition, *Sergentomyia schwetzi* sandflies have been identified in all sandfly pools positive for the newly discovered viruses except for the EMBV-positive pool SP394. Interestingly, *Sergentomyia schwetzi* sandflies have not yet been reported to be associated with phleboviruses. A study including the experimental infection of *Sergentomyia schwetzi* with the mosquito-borne phlebovirus Rift Valley fever virus resulted in a low susceptibility of these sandflies to the virus (26). However, the confirmation of *Sergentomyia schwetzi* sandfly association with maintenance of BGRV, EMBV, KBGV, NPV, and PERV requires further studies, including the detection and replication of these viruses in single sandfly specimens.

In phylogenetic analyses, BGRV, EMBV, and PERV form a sister clade to Dashli virus, Toros virus, and sandfly fever Sicilian virus. Dashli virus has been detected in *Sergentomyia* sp. and Phlebotomus papatasi collected in Iran (15), whereas Toros virus has been detected in sandfly specimen from Turkey belonging to the species Phlebotomus perfiliewi and Phlebotomus tobbi (16). However, Corfou virus, which belongs to the species *Toros phlebovirus*, has been detected in *Phlebotomus major* collected on the eponymous Greek island in the Mediterranean Sea (27). Phlebotomus papatasi has been widely acknowledged as the vector for sandfly fever Sicilian virus, although detection in a variety of *Phlebotomus* species has been reported (1). Interestingly, Phlebotomus papatasi, including the main vector of sandfly fever Sicilian virus, is not known to occur in Kenya (24), favoring speculations that other species could be involved in the transmission of BGRV, EMBV, and PERV (24). Toscana virus-related viruses, sandfly fever Naples virus-related viruses, and Massilia virus-related viruses have been detected in *Phlebotomus perfiliewi* and Phlebotomus perniciosus sandflies. A recent study suggested that the spectrum of competent sandfly vector species for Toscana virus-related viruses is broader than previously thought and includes Phlebotomus longicuspis, Phlebotomus sergenti, Phlebotomus tobbi, Phlebotomus neglectus, and Sergentomyia minuta (28). Rodents have been found to be infected with Gordil virus and Saint Floris virus, although the associated sandfly vector remains elusive (13). These two viruses together with KBGV (reported in this study) have been detected exclusively in Africa. Our phylogenetic analyses placed them in a basal position to the clade of Toscana virus-related viruses, sandfly fever Naples virus-related viruses, and Massilia virus-related viruses, which are present in the Mediterranean, western Asia, and the Indian subcontinent. These findings suggest that the common ancestor of this clade occurred in Africa.

The detection of four highly diverse novel phleboviruses distantly related to the sandfly fever Sicilian and sandfly fever Naples serocomplexes implies that sandfly-borne infections and associated diseases contribute to the health burden in Kenya. Since these viruses were found in a relatively small number of sandflies (*n* = 3,954) originating from a restricted ecology of the Kenyan Rift Valley, the presence of additional sandfly-borne phleboviruses in Kenya and elsewhere in sub-Saharan Africa is highly likely. Taken together with our previous discovery of NPV, our findings represent the southernmost detection of sandfly-associated phleboviruses of potential public health significance in the Old World. Beside mosquitoes and ticks, sandflies should be included in arbovirus surveillance programs focused on epidemic preparedness in Kenya and beyond.

## MATERIALS AND METHODS

### Sandfly collection.

Sandflies were collected in two villages, Ntepes and Kapkuikui of Marigat subcounty, Baringo County, Kenya, in 2015 and 2016 using light-emitting diode (LED) CDC light traps emitting different wavelengths of light (BioQuip, Rancho Dominguez, CA, USA). Traps were placed about 1 m aboveground in and around households and operated for 12 h from dusk until dawn. Adult sandflies were recovered from the field immediately after sunrise, immobilized using triethylamine, transported in liquid nitrogen to the laboratory at the International Centre of Insect Physiology and Ecology (ICIPE), and stored at −80°C until further processing.

### Sandfly RNA extraction and pan-phlebovirus PCR screening.

Sandflies were organized into pools of 10 individuals each, according to collection date and location. Pools were homogenized in 500 μl phosphate-buffered saline (PBS) (Thermo Fisher Scientific, Waltham, MA, USA) using ceramic beads and a SpeedMill Plus (Analytik, Jena, Germany). A 50-μl portion of cleared supernatant from each of 10 pools was used to generate superpools, of which 140 μl was used for RNA extraction using the QIAamp viral RNA minikit (Qiagen, Hilden, Germany). Random hexamer-primed cDNA was synthesized using the SuperScript III RT system (Invitrogen, Karlsruhe, Germany) according to the manufacturer’s instructions, and superpools were tested for phleboviruses as described earlier (29). Obtained sequences were analyzed using Geneious R9.1 (30) and compared to the GenBank database (www.ncbi.nlm.nih.gov/genbank/).

### Real-time RT-PCR screening.

Virus-specific quantitative real-time RT-PCR assays were established for BGRV (forward, 5′-TGAAGCCTGAGTCAAGCCAC; reverse, 5′-CATCATCATCAGACGGGAAGC; probe, 5′-6-carboxyfluorescein [FAM]-AGACATGATGCAGGGTTCAGA), EMBV (forward, 5′-TGAGTCCAGTTCTAAGGTTGTC; reverse, 5′-TCATCATCTGACGGGAAGCTG; probe, 5′-6-FAM-TGACATGATGCAGGGCTCAG-Iowa black fluorescent quencher [IBFQ]), KBGV (forward, 5′-GGAGCTGATGAAAGAATTGTACAATC, reverse, 5′-GGAATTCGTGTCTTGTTGGAGG, probe, 5′-6-TTCTTCGACTCGGACACACTATGCAA-IBFQ), and PERV (forward, 5′-ACACCAAGAGTTTATCAGGACTATG, reverse, 5′-CATCTGATCCCTGCATCATGTC, probe, 5′-6-FAM-ACCAGAAATGAGCAACAGAGT-IBFQ) and used to test individual pools of virus-positive superpools and cell culture supernatants. The assay used for the detection of NPV was described earlier (19).

### Genome sequencing and analyses.

Sample libraries were prepared from RNA of phlebovirus-positive sandfly pools using the KAPA HyperPlus kit (Roche, Penzberg, Germany) and sequenced using the Illumina MiSeq HTS platform as described earlier (29). After demultiplexing, the paired end reads were filtered using AdapterRemoval 2.2.2 (31), trimming read end N bases and read end bases with a quality score of 2 or lower, as well as reads shorter than 30 nucleotides. Paired reads were merged using FLASH v1.2.11 (32), and all reads were further filtered by mapping against the reference genome of Aedes albopictus using bwa mem 0.7.15-r1140 (33). For the filtered reads, a DIAMOND 0.9.23 (34) search was performed against the Reference Viral Database 14.0 (35) (downloaded on 12 December 2018) and against the NCBI viral protein RefSeq database (36) (downloaded on 17 July 2018). Reads mapping against phlebovirus S and M segments were identified. Together with the initial RdRp screening fragment, the sequences were subjected to an iterated reference mapping of filtered HTS reads to the respective sequence using Geneious mapper (30). Genome gaps and ends were amplified by conventional seminested RT-PCR as described earlier (29). PCR products were Sanger sequenced. Full genomes were analyzed using Geneious R9 (30). Geneious-implementing InterProScan (37) was used to predict transmembrane domains and posttranslational cleavage sites of the GPC. N-glycosylation sites of the M segment were predicted using the NetNGlyc v1.0 server (http://www.cbs.dtu.dk/services/NetNGlyc/).

### Genotyping of sandflies and blood meal analysis.

For sandfly species identification, the RNA extracts of virus positive sandfly pools containing coeluted sandfly DNA were subjected to amplification of the invertebrate cytochrome *c* oxidase subunit I (*COI*) gene as described earlier (38). PCR products were cloned into the pCR4-TOPO vector, and 10 clones were Sanger sequenced using vector primers. After vector trimming, sandfly *COI* sequences were compared with the GenBank database, applying species-level demarcation (≥98%) as suggested by Valinsky and colleagues (39).

Blood meal analyses of virus-positive pools were performed as described by Alcaide et al., targeting the vertebrate cytochrome *c* oxidase subunit I (*COI*) in coeluted DNA from RNA extracts (40). PCR products were Sanger sequenced and compared with GenBank and BOLD databases using the criteria mentioned above.

### Sequencing of N genes.

Nearly complete coding sequences of the nucleocapsid (N) genes were amplified from each detected virus strain by RT-PCR using gene-specific primers. PCR products were sequenced by Sanger sequencing.

### Phylogenetic analysis.

Amino acid sequences of the L, Gn, Gc, and N proteins were aligned using the MAFFT E-INS-I algorithm (41). Phylogenies were inferred using PhyML with the LG substitution model as implemented in Geneious R9 and confidence testing over 1,000 bootstrap replicates (42).

### Recombinant immunofluorescent assays.

Synthesized FLAG-tagged full-length N genes of BGRV, EMBV, KBGV, and PERV were purchased from Integrated DNA Technologies (Leuven, Belgium) and cloned into the pCG1 vector. VeroE6 cells were transfected with the respective construct, and 1.25 × 10^4^ cells were spotted onto multitest cover slides. Cells were fixed using ice-cold acetone-methanol (1:1 ratio). In addition, commercial multitest cover slides from Euroimmun (Lübeck, Germany) containing cells infected with sandfly fever Sicilian virus (SFSV), sandfly fever Naples virus (SFNV), and Toscana virus (TOSV) were used. Humanized rabbit anti-TOSV N monoclonal antibodies, rabbit anti-FLAG antibodies, and human serum samples reactive against SFSV, SFNV, and TOSV were diluted in sample buffer (Euroimmun, Lübeck, Germany) and applied on the multitest cover slides coated with transfected VeroE6 cells. Secondary fluorophore-labeled goat anti-rabbit IgG–Alexa Fluor 488 and goat anti-human IgG–Cy2 antibodies (Dianova GmbH, Hamburg, Germany) were applied after washing steps to the corresponding samples, and multitest cover slides were examined using a fluorescence microscope.

### Virus isolation trials.

Grivet (Chlorocebus aethiops) VeroE6 cells, mosquito (Aedes albopictus) C6/36 cells, rhesus macaque (Macaca mulatta) LLC-MK2 cells, sandfly (Lutzomyia longipalpis) LL-5 cells, sandfly (Phlebotomus papatasi) PP-9 cells, and Syrian hamster (Mesocricetus auratus) BHK-21 cells were used to perform virus isolation trials from diluted sandfly homogenates (1:10 and 1:100) as described previously (29). Cells were observed regularly for the occurrence of a cytopathic effect. Infectious supernatants were passaged three times on fresh cells after 7 days postinfection, and virus replication was measured by virus-specific real-time RT-PCR (see above).

### Screening of human serum samples.

Human serum samples (*n* = 244) were collected from patients with fever of unknown origin hospitalized in Marigat subcounty. A 5-μl portion of individual sera was used for RNA extraction using the QIAamp viral RNA minikit (Qiagen, Hilden, Germany). cDNA was synthesized using random hexamer primer and the SuperScript III RT system (Invitrogen, Karlsruhe, Germany). Virus-specific quantitative real-time RT-PCR assays as mentioned above were used to test individual extracted RNA for direct evidence of the viruses in human blood.

### Ethical considerations.

Approval for the study was granted by the Scientific and Ethical Review Unit of the Kenya Medical Research Institute (SSC protocol number 1560).

### Data availability.

Coding-complete genomes of Bogoria virus, Kiborgoch virus, Perkerra virus, and Embossos virus have been deposited in GenBank under accession numbers MT270825 to MT270836 and those of NPV under accession numbers MT625964 to MT625966. Partial N-gene sequences of additional strains of Embossos virus and Perkerra virus are deposited under accession numbers MT625967 and MT625968. The *COI* sequences of sandflies and blood meal sources have also been deposited in GenBank under accession numbers SAMN15848018, SAMN15848019, SAMN15848020, and SAMN15848021. HTS data were deposited in the Sequence Read Archive (SRA) under accession no. PRJNA657829.
